# The ultrafiltration coefficient: this old ‘grand inconnu’ in dialysis

**DOI:** 10.1093/ndt/gft493

**Published:** 2013-12-19

**Authors:** Alain Ficheux, Claudio Ronco, Philippe Brunet, Àngel Argilés

**Affiliations:** 1 RD – Néphrologie and Groupe Rein et HTA, EA3127, Institut Universitaire de Recherche Clinique IURC - UM1, Montpellier 34090, France; 2 Department of Nephrology Dialysis and Transplantation, International Renal Research Institute (IRRIV), San Bortolo Hospital, Vicenza 36100, Italy; 3 Service de Néphrologie, Hôpital de La Conception – Université Aix-Marseille, Marseille 13005, France; 4 Centre de dialyse de Sète, Néphrologie Dialyse St Guilhem, Sète 34204, France

**Keywords:** convection, dialysis, ultrafiltration coefficient, water permeability

## INTRODUCTION

Although a wide range of physical principles capable of separating different solutes exist in biochemistry (such as affinity, or size as well as charge retaining columns and others), the removal of uraemic solutes has been almost exclusively performed up to the present with membrane-based systems. Sir Thomas Graham, in the second half of the 1800s, defined the method of separating various fluids by diffusion through a membrane with the term ‘dialysis’[1]. Galen in the second century of our era already claimed that the skin resembles a sieve and ‘sweating purifies the body, … by low-effort exercise, baths and the summer heat’ [*De Symptomatum Causis Libri III, Claudii Galeni Opera Omnia (II)*][2], and ancient Romans used the skin as a natural membrane to rid their bodies of poisonous urinal substances in the Therms and public baths. Well into the 20th century, artificial kidneys, based on membrane devices were adopted and the pioneer work by Abel, Rowntree and Turner [3], as well as that of Haas [4], was followed by the rotatory drum dialyser of Willem Kolff [5] and the vertical drum one of Nils Alwall [6]. Finally, the hollow fibre dialysers gained adepts and a widespread use of cuprophane membranes for a very long period of time (from the 1970s to the 1990s) has been followed by the introduction of high-flux membranes that have invaded most of the dialysis units worldwide to the present.

It became quite clear from the very beginning that membranes differ in their clearance capacities of the different solutes, basically depending on thickness and pore size. However, increasing the pore size and reducing thickness is almost forcedly associated to a water permeability increase. The open dialysate circuit settings used during the era of low-permeability membranes had to be secured by the addition of ultrafiltration controllers, which closed the dialysis circuit [7], and are mandatory when using high-flux membranes (highly permeable to water) particularly if convective techniques are utilized.

Defining water permeability of a dialyser was considered important from the beginning and is even more important with the high-flux dialysers. Water permeability of a dialyser was defined by its ultrafiltration coefficient, which is displayed in the notice of the given dialyser.

The coefficient of ultrafiltration (*K*_UF_) was first defined by the amount of fluid (*V*) in mL crossing the dialyser membrane per time (*T*) in hours and pressure (*P*) in mmHg:

$$K_{\mathrm{UF}}=\frac{V}{T\times P}$$

The perception that renal physicians have of *K*_UF_ has changed over time. Senior nephrologists considered *K*_UF_ as a constant and took it into account in dialysis prescription in the low-permeability era [8]; it was common to hear comments on the different *K*_UF_ or ‘slope’ of one dialyser in regard to another one in clinics and the consequences that this might have to the treatment and to the patient. Among senior physicians, only those particularly interested on the topic knew that *K*_UF_ was not always a constant as its value may vary over a certain range of filtration rate. Young nephrologists, who have only lived the ultrafiltration controller era, have just ignored *K*_UF_. They simply did not need it.

Nevertheless, the importance of *K*_UF_ of the early times has remained in many aspects, including the approval of new devices by the regulatory agencies such as the US Food and Drugs Administration (FDA) [9] or its equivalent in Europe, the European Medicines Agency (EMA), a prerequisite to use them in clinics in all these countries. Indeed, the recent randomized, controlled trials on haemodiafiltration [10–12] and particularly that of Maduell *et al*. [12] providing evidence that high convective volume may improve survival has given a renewed protagonism to *K*_UF_, as it influences the convective capacities of the dialysis setting. *K*_UF_ remains, though, the old ‘grand inconnu’. In the present editorial comment, we want to present a refurbished *K*_UF_ to society, going in-depth into the factors influencing *K*_UF_ and its calculation, and then coming back with as simple as possible methods to obtain it for easy clinical use.

## DO WE KNOW *K*_UF_?

*K*_UF_ is defined by the American National Standards Institute (ANSI) as the permeability of a membrane to water, generally expressed in millilitres per hour per millimetre of mercury (ANSI/AAMI/ISO 8637:2010)[13]. However, this definition concerns the permeability of the membrane and not that of the device: the dialyser.

### General formula for the determination of the *K*_UF_ of a membrane

The simplified calculation of a membrane's *K*_UF_ is based upon Darcy's law: ‘The filtration flow (*Q*_UF_) is proportional to the pressure difference between the two faces of the filter (Δ*P*) and to its surface (*S*)’. This law to be fulfilled requires the membrane being homogeneous without deposits, a steady pressure throughout the membrane surface and the fluid's viscosity being also constant.

The simplified formula is:

$$Q_{\mathrm{UF}}=K_{\mathrm{UF}}\text{s}\times \Delta P\times S$$

where *K*_UF_s is the ultrafiltration coefficient of the membrane per surface unit; Δ*P* is the pressure difference between the two faces of the membrane; *S* is the surface of the membrane.

The ultrafiltration coefficient of the filtrating device, in our case, the dialyser is

(1) $$K_{\mathrm{UF}}=K_{\mathrm{UF}}\text{s}\times S\text{,}$$

which following Darcy's law can be defined as follows:

(2) $$K_{\mathrm{UF}}=\frac{Q_{\mathrm{UF}}}{\Delta P}$$

where Δ*P* is the pressure difference between the two faces of the membrane; Δ*P* is the resultant of the hydrostatic pressure and the pressure induced by the constituents of the fluid (osmotic and oncotic pressures).

#### Measurement of the *K*_UF_ of a membrane system with an open ultrafiltrate circuit

The requirements defined by the Association for the Advancement of Medical Instrumentation (ANSI/AAMI RD16:1996), on which the FDA based its exigencies to homologate a dialyser up to 2010 include the description of the *K*_UF_ *in vivo* and *in vitro* with a limited variability in its values (10% as reported by Keshaviah *et al.*, 17% in most of the dialysers and 20% mandatory). They proposed the measurements of *K*_UF_ to be performed without circulating dialysate following Keshaviah's method [14] which was set in an open dialysate side circuit and assuming a positive filtration from the blood side to the dialysate side all throughout the dialyser. They fixed TMP at 0, 100 and 300 mmHg and the maximum tolerated by the membrane and collected the ultrafiltrate; they considered *K*_UF_ as the slope of the regression line of TMP over *Q*_UF_. The TMP at *Q*_UF_ = 0, TMP0 is the value accepted as equal to the amount of pressure that opposes the production of fluid and is taken as equal to the oncotic pressure *π*. Although *π* will change with increasing filtration, it is considered constant over the measured range and the general formula [2] is often amended as follows [15]:

(3) $$K_{\mathrm{UF}}=\frac{Q_{\mathrm{UF}}}{\text{TMP}-\pi }$$

**FIGURE 1:**
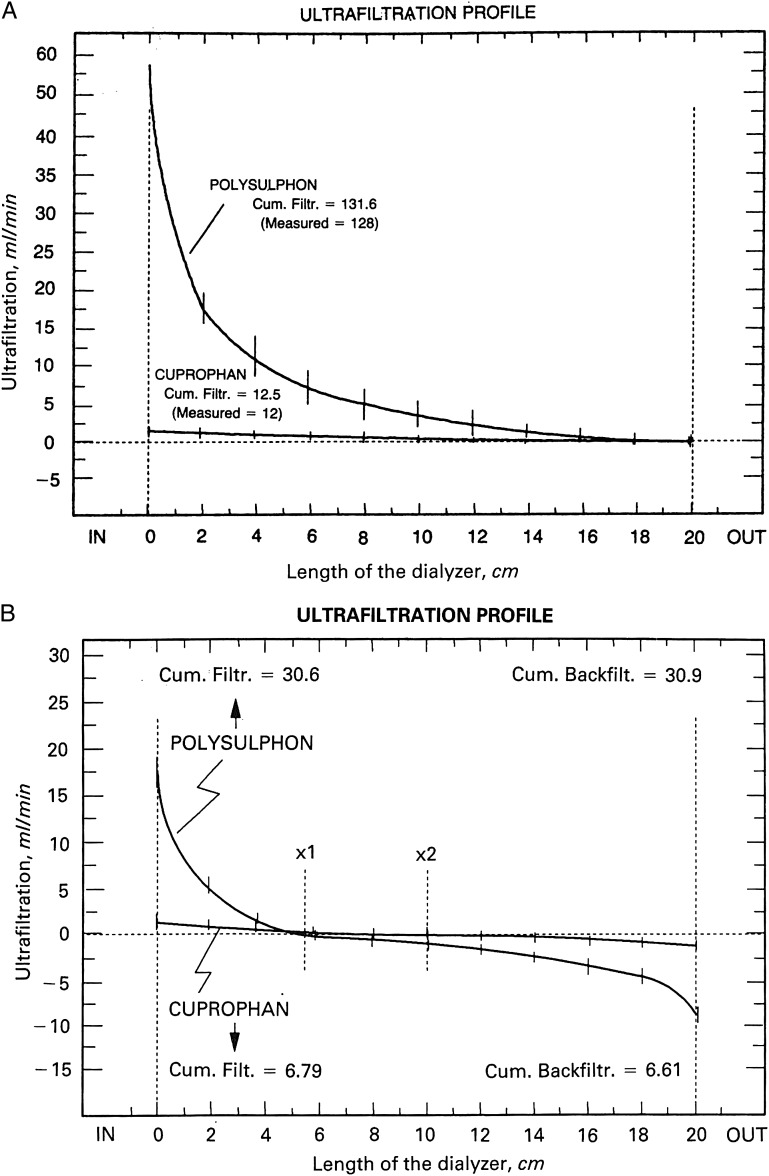
Ultrafiltration profiles derived from albumin concentration along the length of the dialysers. (**A**) Maximal ultrafiltration is observed at the proximal end of the dialyser with a subsequent decrease to zero at the distal end. (**B**) Maximal ultrafiltration is observed at the proximal end of the dialyser with a subsequent decrease to zero at different points of the polysulphone (×1) and cuprophane (×2). From these points, backfiltration begins reaching its maximum at the distal end of the dialyser. Despite different profiles are observed, cumulative ultrafiltration and cumulative backfiltration are equal. (Modified from ref. [19], reprinted by permission from Macmillan Publishers Ltd).

In this setting, the filtration is always from the blood side to the external or dialysate side for the whole length of the dialyser's fibres (see Figure 1A) and it was well adapted to the low-permeability dialysers.

#### Measurement of the *K*_UF_ of a membrane in a system with a closed ultrafiltrate circuit

To determine the *K*_UF_ of a high-permeability dialyser, the AAMI recommends the use of an ultrafiltration setting with an ultrafiltration pump to regulate the *Q*_UF_ and to measure *Q*_UF_ over the manufacturer's specified range; this pump closes the ultrafiltrate circuit. As in the open system, *K*_UF_ is calculated as the slope of the regression line between *Q*_UF_ and TMP, taking oncotic pressure (*π* determined as the value at the origin of the regression line) into account.

In haemodialysis, with the advent of the high-permeability membranes and the need for controlling ultrafiltration rates, the dialysate side circuit was also closed so that the total ultrafiltrered volume was controlled. By doing so, particularly in the high-permeability dialysers, the filtration of fluid inside the dialyser is both directions: from blood to dialysate and also from the dialysate side to blood to obtain a resultant *Q*_UF_ programmed and no extra ultrafiltration flow [16, 17]. The filtration from the dialysate side to the blood is called ‘backfiltration’ and the point where filtration changes direction (see Figure 1B) may move alongside the membrane of the dialyser [18]. In the closed setting, not only the effective surface of net filtration and that of net backfiltration may change, but blood viscosity and pressures, including hydrostatic and oncotic pressure, do change. Indeed, in this setting, the linear equation to determine *K*_UF_ [3] does not apply [19].

## GOING TO THE ENTRAILS OF THE *K*_UF_: WHAT IS OCCURRING INSIDE THE DIALYSER?

In the 1990s, Ronco *et al.* nicely assessed the filtration within the dialyser by colorimetric and scintigraphic methods [19] and established the crossing point of the two flows: filtration and backfiltration. They were able to define both filtration flows and concluded that linear models are not adequate to predict the water kinetics across dialysis membranes [19].

The filtration flows have a characteristic *K*_UF_ within the dialyser which follows the following formula:

$$Q_{\mathrm{UF}}=\int \int_{0}^{S}\Delta P\cdot K_{\mathrm{UF}}\cdot \text{d}S$$

It is of note that both Δ*P* and *Q*_UF_ vary alongside the dialyser fibres under the influence of plasma protein concentration and oncotic pressure, haematocrit and blood viscosity. The integral takes into account these variations at every point. However, the actual value of each of these at every point of the membrane remains very difficult to determine and submitted to errors. When Δ*P* is <0, the filtration flow is from the dialysate side to the blood side (backfiltration).

## HAVING A LOOK OUTSIDE THE DIALYSER

### The global *K*_UF_ or *_G_K_D_*_-UF_

Given the difficulty in determining *K*_UF_ at every point alongside the dialyser, new approaches have appeared to simplify and eliminate the probability of errors. This is the approach taken when measuring the global *K*_UF_ of the system [20] that in the present report is referred to as *_G_K_D_*_-UF_ (*_G_* = for global; *K* = for coefficient; *_D_* = for dilaysis; and _UF_ = for ultrafiltration).

*_G_K_D_*_-UF_ is the resultant *K*_UF_ obtained with the resultant *Q*_UF_ and the resultant pressures in the system. It does not rely on every point measurements alongside the membrane of the dialyser but on the global values. It is measured as follows:

$${}_{G}K_{D-\mathrm{UF}}=\frac{Q_{\mathrm{UF}}}{\text{TMP}}$$

where *Q*_UF_ (in mL/h) is the total ultrafiltration flow given by the dialysis machine. It represents the net flow after including filtration and backfiltration.

TMP (in mmHg) is the resultant pressure of the system incorporating the measurements of pressures at the different sides of the system (blood inlet, blood outlet, dialysate inlet and dialysate outlet). It is a simple measure which encompasses all the modifications occurring inside the dialyser (including viscosity induced resistance to filtration flow or oncotic pressure variation), without knowing their individual values, into a global measurement.

Since the measures are taken outside the dialyser in a particular day with a particular patient, the obtained values correspond to the global *K*_UF_*s* of the system that day for that patient. *_G_K_D_*_-UF_ is not the *K*_UF_ of a membrane or even of a dialyser, which have to be mandatorily obtained with values of that membrane alongside its length.

In our previous study, we called the *K*_UF_ obtained with the external measures, ‘*K*_UF_ of the whole dialysis system’ [20]. We purposely decided to call it *_G_K_D_*_-UF_ in the present report in order to differentiate it from the other *K*_UF_*s*, such as those already commented and avoid any confusion.

#### *_G_K_D_*_-UF_ variation over *Q*_UF_

**FIGURE 2:**
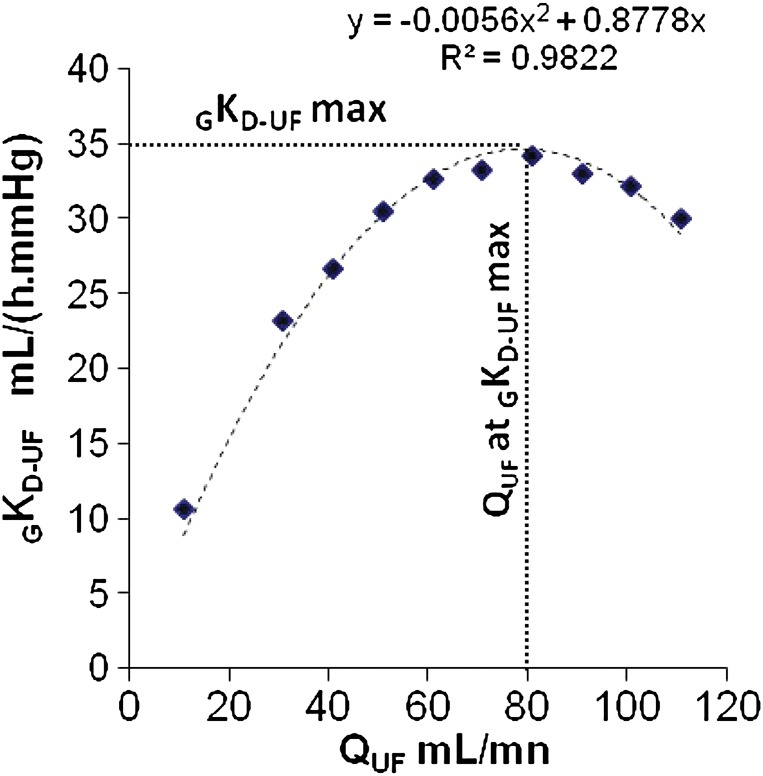
Determining the *_G_K_D_*_-UF_ over a range of *Q*_UF_. An example of *_G_K_D_*_-UF_ determination at the bedside at the initiation of the dialysis procedure is presented. The correlation score (*R*²) and the regression line are given. (Note that *R*² is close to 1). The value of *_G_K_D_*_-UF_-max is plotted on the *y* axis just over 35 mL h^−1^ mmHg^−1^. The *Q*_UF_ rate at which *_G_K_D_*_-UF_ max is observed is plotted on the *x* axis (around 80 mL/min). The concept of GKD-UF has been reported in ref. [20].

When controlling *Q*_UF_ over a wide range and measuring TMP, the obtained values of *_G_K_D_*_-UF_ follow a parabolic function (Figure 2). Therefore, *_G_K_D_*_-UF_ is not a constant; it varies with increasing *Q*_UF_, increasing first, up to the vertex of the parabola or maximum value of *_G_K_D_*_-UF_ and decreasing thereafter if *Q*_UF_ is still increased.

The parabolic model of *_G_K_D_*_-UF_ variation differs from the linear model of *K*_UF_ over *Q*_UF_. We have already commented that the values inside the dialyser are difficult to measure and do not follow simple laws. Already from the early period of low permeability and open dialysate side, some attempts have proposed to simplify these measurements. One of them is to subtract the value of oncotic pressure, obtained with the value of *x*-axis at the origin of the regression line (*y* = 0) as commented for the Keshaviah's method, in the determinations of *K*_UF_. This approach which could be of help in the open settings is no longer applicable to closed systems, where oncotic pressure increases within the dialyser until the crossing point of fluxes and decreases thereafter. Thus, it would not be sound to subtract a constant value, which would become arbitrary, from the measured TMP, as we know that both the crossing point and oncotic pressure change by changing *Q*_UF_.

#### Can we explain why *_G_K_D_*_-UF_ variation over *Q*_UF_ follows a parabolic function?

After having seen the work by Ronco *et al.* on the filtration fluxes of two opposite directions alongside the dialyser and given that the *x* point where filtration fluxes change direction may move alongside the dialyser, one could speculate that the parabolic shape of the *_G_K_D_*_-UF_ over *Q*_UF_ is the consequence of shifting the *x* point within the dialyser. When increasing *Q*_UF_*s* are solicited from the system, an increase in hydrostatic pressure will follow and the filtrating surface will increase. As the total surface is unextendable, the backfiltrating surface will decrease. *K*_UF_ is directly proportional to the surface (see formula [1]), and as a consequence, it will increase. It will increase until the minimal backfiltrating surface will be reached, and most of surface of the dialyser will be filtrating from the blood side to the dialysate side. Beyond this point of *Q*_UF_, if a further increase of *Q*_UF_ is requested, to obtain a differential increase in *Q*_UF_, a more important increase of pressure will be required and, as a consequence, the *_G_K_D_*_-UF_ of the system will start decreasing, drawing then a parabolic shape, which will be indeed the result of the increase in oncotic pressure, but no only; it might be influenced by haemoconcentration, membrane modifications and other factors.

## TO THE POINT: *K*_UF_ DOES IT MATTER IN NOWADAYS DIALYSIS SYSTEMS?

As dialysis is based on a membrane system, the driving forces of the system do matter as also do the limiting factors of the membrane system, such as the diffusion constants driving clearance of the different solutes (width of the membrane, improvement in the thickness and the nanotechnology). Hydraulic permeability or *K*_UF_, the main factor driving convection is therefore of outmost importance.

## CONCLUSIONS

Understanding what is occurring inside the dialyser is important and we know how difficult it is to determine every factor influencing efficacy of a dialysis system. In a moment that convection is gaining the protagonist place in dialysis, *K*_UF_ is doing its come back to the scene. Simple methods to quantify the hydraulic permeability of a given system, such as *_G_K_D_*_-UF_ should be welcomed as (i) they are informative of the conditions of the system, (ii) they are not incompatible with the assumptions and formulas but simplify them by measuring a global component and (iii) they represent an objective parameter easily available to drive convection with a better understanding of the constraints the fluid (blood) is submitted to in the system.

## CONFLICT OF INTEREST STATEMENT

A.F. and À.A. are employees of RD Néphrologie, a spin-off of the CNRS (France), owner of the patent Number WO 2010 040927 protecting the rights on the exploitation of *_G_K_D_*_-UF_. C.R. and P.B. have declared no conflict of interest. Funding to pay the Open Access publication charges for this article was provided by B BRAUN Avitum (Melsunguen, Germany).
